# Successful interventions on an organisational level to reduce violence and coercive interventions in in-patients with adjustment disorders and personality disorders

**DOI:** 10.1186/1745-0179-4-27

**Published:** 2008-11-17

**Authors:** Tilman Steinert, Frank Eisele, Ulla Goeser, Stefan Tschoeke, Carmen Uhlmann, Peter Schmid

**Affiliations:** 1 Centre for Psychiatry Weissenau, Clinical Department, Germany; 2 Department of Psychiatry I, Ulm University, Ravensburg, Weingartshofer Str. 2, 88214 Ravensburg, Germany; 3 Centres for Psychiatry Südwürttemberg, Data Management, Germany; 4 Zentrum für Psychiatrie Weissenau, Dept. Psychiatry I, Ulm University, Postfach 2044, D 88190 Ravensburg-Weissenau, Germany

## Abstract

**Background:**

Self-directed and other violence as well as subsequent coercive interventions occur in a substantial proportion of patients with personality disorders during in-patient treatment. Different strategies may be required to reduce coercive interventions for patients of different diagnostic groups.

**Methods:**

We specialised one of our acute admission wards in the treatment of personality disorders and adjustment disorders (ICD-10 F4 and F6). Patients are not transferred to other acute wards in case of suicidal or violent behaviour. Violent behaviour and coercive interventions such as seclusion or restraint were recorded in the same way as in the rest of the hospital. We recorded the percentage of subjects affected by diagnostic group and average length of an intervention in the year before and after the change in organisational structure.

**Results:**

The total number of coercive interventions decreased by 85% both among patients with an F4 and those with an F6 primary diagnosis. Violent behaviours decreased by about 50%, the proportion of involuntary committed patients decreased by 70%.

**Conclusion:**

The organisational change turned out to be highly effective without any additional cost of personnel or other resources.

## Background

In the 21st century, seclusion and restraint are still widespread practices in psychiatric hospitals worldwide. The most frequent reason for such coercive interventions is overt or imminent violent behaviour, followed by self-directed aggressive behaviour [1-6]. However, in the last ten years in many countries, initiatives emerged to prevent violence and to decrease or ban the use of coercive measures [5,7,8]. The focus of such interventions has primarily been training in de-escalation strategies [9,10], followed by initiatives to change policy, culture, and staff attitudes [11]. In Germany, about 9% of in-patients are affected by seclusion or restraint, with the most affected group being demented patients who are restrained to their beds during night-time in order to prevent falls [12]. Whilst training and changes of attitudes can be applied to all hospital staff, different types of specialised interventions are required for different groups of patients. For example, coercive interventions among demented patients can be reduced by the use of hip protectors or alarm mattresses [13], for patients with psychotic disorders regular assessments of violence risk and structured care plans are being seen as adequate interventions [14,15].

In a survey of coercive measures in about 36.000 admissions to 10 hospitals in 2004 [12], our hospital had a relatively high percentage of coercive measures in the diagnostic group ICD-10 F6 (personality disorders) compared to other hospitals. 15.8% of patients with F6 disorders had been affected by seclusion or restraint in comparison to an average of 9.4% across the ten hospitals. Thus, interventions adequate for this group were required. Before the survey, patients with personality disorders and adjustment disorders had been treated in keeping with widespread practice in Germany. This practice suggests that such patients can receive treatment on specialised psychotherapy units or hospitals, but in case of suicidal or violent behaviour they are referred to mostly locked acute psychiatric wards, where they are treated among predominantly psychotic patients. The most frequent cause for admission of patients with so-called adjustment disorders (ICD-10 F 43.2) is a recent history of a suicide attempt. Many of these patients suffer from personality disorders and may also have posttraumatic stress disorder. The admission is frequently not officially involuntary but is initiated with considerable pressure from referring doctors or relatives. In the environment of an acute admission ward, which is often characterised by an atmosphere of tension and strange behaviours, these patients frequently do not consent to treatment and exhibit further violent or suicidal behaviour. Because training for staff and specific guidelines for the management of violent behaviour were already available at the hospital, we decided to intervene on the level of hospital organisation and established a specialised ward for this group of patients. A crucial prerequisite, however, was, that any change had to be cost-neutral internally (staff resources) and externally (number of hospital days).

## Methods

### The concept

While until 31.10.2005 patients with ICD-10 F4 and F6 disorders were referred to one of four acute admission wards in case of a crisis, from the 1.11.2005 onwards they were referred nearly exclusively to one of the four former acute admission wards with 18 beds now became specialised in the treatment of patients with personality disorders and adjustment disorders. This ward was named "crisis intervention ward". A considerable proportion of the patients have concomitant diagnoses of substance abuse. However, a primary diagnosis of substance dependency (ICD-10 F 1x.2), a psychotic or an affective disorder (ICD-10 F2, F3) is an exclusion criterion for this ward. The team consists of two specialised doctors for psychiatry and psychotherapy, sometimes a psychologist trainee, a part-time social worker and 13.5 full-time nurses. Patients can be treated on a voluntary or, if necessary, involuntary basis. The door is mostly open, but can be locked. Patients are offered three different paths ("modules") of therapy and can to a certain degree decide for themselves about the procedure:

- Module "crisis": frequent observation (but usually not 1:1), no responsibilities, prohibition to leave the ward, therapeutic conversations with therapists only about their suicidality or violence risk; if necessary, even seclusion and restraint can be applied.

- module "therapy": comprises psychotherapeutic sessions, education about personality disorders, dialectic behavioural therapy in groups [16], occupational therapy and freedom to leave the ward, accompanied by increased responsibilities. To enter the module "therapy", patients have to sign a treatment contract.

- module "discharge":contains predominantly discussions with the social worker about expected conditions of life after discharge.

### Data recording and processing

Diagnoses are routinely made using ICD-10 criteria by at least two specialised psychiatrists. If necessary, further co-morbid diagnoses are made. Coercive interventions are recorded in the electronic charts with respect to cause, legal prerequisites, kind of measure, duration and participating persons. This system is established on all wards of the hospital. Further details of definitions, key measures, validity and ethical aspects have been described extensively elsewhere [12].

Suicidal and aggressive behaviours were recorded in the hospital's electronic "basic documentation" as suggested by the German Psychiatric Association DGPPN [17]. The following items were used for this purpose: Violent threats, violence against objects, violence against persons, suicide attempt during hospital stay, and involuntary commitment under two different laws regulating this procedure in Germany: Law Regulating Custody (German BGB) or public commitment law (German UBG). Data for each of these items are available dichotomously (yes/no). Whilst data on coercive interventions were available for all patients, the data from the basic documentation were missing for some patients. Complete data sets were available for 230 out of 287 discharged patients (80.1%) in the year before and for 279 out of 301 (92.7%) discharged patients in the year after the opening of the crisis intervention ward. There was no systematic bias in missing data, thus the results can be looked upon as representative. The slightly reduced number regarding measurements of violence is indicated in the respective items in tables 1 and 2.

**Table 1:** Frequency of aggressive behaviour and coercive measures before and after conceptual change in patients with anxiety and adjustment disorders [ICD-10 F 4]

	1.11.2004–31.10.2005	1.11.2005–31.10.2006	Change
Treatment episodes	N = 160	N = 154	-3.8%
affected by mechanical restraint	4.3%	0.6%	-86%
affected by seclusion	5.0%	2.6%	-48%
Mean duration of mechanical restraint	13.6 h	2.0 h	-85%
Mean duration of seclusion	2.8 h	4.3 h	+ 54%
Mean number of any kind of coercive interventions per affected patient	3.4	1.4	-58%
Total number of coercive interventions	48	7	-85%
Suicide attempt^1^	19.2%	19.7%	+3.6%
Violent threat^1^	9.2%	4.2%	-54%
Violence against objects^1^	3.8%	2.7%	-29%
Violence against persons^1^	7.7%	1.4%	-81% [p < .05]
Involuntary commitment^1^	3.8%	0.6%	-84%

^1 ^reduced n available for this item: 130 before/147 after intervention

**Table 2:** Frequency of aggressive behaviour and coercive measures before and after conceptual change in patients with personality disorders [ICD-10 F 6]

	1.11.2004–31.10.2005	1.11.2005–31.10.2006	Change
Treatment episodes	N = 127	N = 147	+ 15.7%
affected by mechanical restraint	6.3%	3.4%	-46%
affected by seclusion	15.0%	2.7%	-82% [p < .001]
Mean duration of mechanical restraint	3.7 h	12.4 h	+ 230% [p < .01]
Mean duration of seclusion	17.0 h	8.1 h	-52%
Mean number of any kind of coercive interventions per affected patient	4.9	2.3	-53%
Total number of coercive interventions	120	17	-86%
Suicide attempt^1^	10.0%	7.6%	-24%
Violent threat^1^	5.0%	4.5%	-10%
Violence against objects^1^	5.0%	4.5%	-10%
Violence against persons^1^	7.0%	3.8%	-45.7%
Involuntary commitment^1^	11.0%	3.8%	-65.5% [p < .05]

^1 ^reduced n available for this item: 100 before/132 after intervention

Medication was given according to clinical symptoms without change in clinical practice over the whole observation period. Medication was not recorded for study purposes.

### Samples and evaluation periods

The evaluation comprised all discharged patients with a primary diagnosis of ICD-10 F4 or F6 in the first year after the change of organisation, 1.11.2005 – 31.10.2006. As a comparison group we used the discharged patients of the same diagnoses in the year before (1.11.2004 – 31.10.2005) (pre-post-design). Patients readmitted in the same period were included twice. Patients with secondary diagnosis F4 or F6 and other primary diagnoses (schizophrenic, affective, or organic disorder, substance abuse) were not included. The evaluation was done not just taking into account the figures from the new crisis intervention ward on ward level but we included figures from the whole hospital since a significant part of patients with F4 and F6 disorders were still treated on other wards after the implementation of the crisis intervention ward. This comprised a considerable number of patients in day-clinic treatment and those who were admitted to other wards if no bed was available on the crisis intervention ward. Had an evaluation been done only on the crisis intervention ward, any effects could been questioned arguing that coercive interventions might have taken place predominantly among those individuals with F4 and F6 diagnosis who had been treated on other wards.

### Statistical methods

Differences in proportions of affected patients were tested by Chi square test or Fisher's exact test in case of cells with numbers < 6, differences in time measurements (hours in seclusion, length of stay) were tested by t-tests. The numbers of coercive measures per patient were compared using the Mann-Whitney U-test. The level of significance was determined as .05.

## Results

Characteristics of the patients treated in the year before and after the implementation of the crisis intervention ward are presented in table 3. The distribution of diagnoses among admitted patients remained unchanged. There was a moderate increase in the percentage of admitted females and a significant increase of length of stay among patients with adjustment disorders. Generally, co-morbidity was rather frequent with at least one secondary diagnosis in about two thirds of the patients treated, both before and after the intervention. Most frequent was a somatic secondary diagnosis (38% of all secondary diagnoses in the year before and 46% in the year after the intervention), followed by substance misuse.

**Table 3:** Patient characteristics

	1.11.2004–31.10.2005	1.11.2004–31.10.2005	1.11.2005–31.10.2006	1.11.2005–31.10.2006
	Adjustment disorders (ICD-10 F4)	Personality disorders (ICD-10 F6)	Adjustment disorders (ICD-10 F4)	Personality disorders (ICD-10 F6)
N	160	127	154	147
% female	55.0%	64.6%	60.4%	76.9%*
Age (mean, years)	37.4	34.4	36.8	33.0
Length of stay (mean, days)	16.3	30.0	23.6*	31.6
Primary diagnosis	Anxiety disorder (F 40.x, F 41.x) 14.4%	Borderline (F 60.30, F 60.31) 70.0%	Anxiety disorder (F 40.x, F 41.x) 14.3%	Borderline (F 60.30, F 60.31) 70.7%
	Obsessive-compulsive disorder (F 42.x) 6.3%	Antisocial, histrionic, narcisstic (F 60.2, 60.4, 60.8) 12.6%	Obsessive-compulsive disorder (F 42.x) 5.8%	Antisocial, histrionic, narcisstic (F 60.2, 60.4, 60.8) 4.1%
	Stress and adjustment disorders (F 43.x) 74.4%	Combined (F 61) 11.8%	Stress and adjustment disorders (F 43.x) 72.7%	Combined (F 61) 19.7%
		Other 5.6%		Other 5.5%
	Dissociative disorder (F 44.x) 1.3%		Dissociative disorder (F 44.x) 1.5%	
	Somatoform disorder (F 45.x) 3.1%		Somatoform disorder (F 45.x) 2.6%	
	Other 2.5%		Other 3.1%	
Secondary diagnosis	65%	68%	60%	65%
Secondary diagnosis substance abuse	24.4%	32.3%	14.3%*	39.4%

* p < .05 sign. difference in comparison with the same diagnostic group in the other year

We found a lower incidence for all outcome measures in the year after the introduction of the crisis intervention ward (see tables 1 and 2) with the exception of the mean duration of mechanical restraint in patients with personality disorders, which had significantly increased,. However, the numbers were small and in the same time the absolute number of applied restraints dropped from 84 to 9, the number of applied seclusions from 84 to 15.

Due to the relatively small numbers of the observed adverse events, in many cases the decrease did not reach significance, but the tendency was the same for all outcomes. Most significantly, the absolute number of interventions dropped to one-seventh, the proportion of patients with violent incidents decreased to one-fifth among patients with adjustment disorders, and the percentage of patients with personality disorders affected by seclusion also dropped to one-fifth. The proportion of involuntary committed patients dropped from 7.0 to 2.1% for the whole sample. The number of suicide attempts remained unchanged.

A further analysis of the distribution of admitted patients in the hospital after introduction of the crisis intervention ward revealed that 55.8% of patients with F4 and F6 disorders were admitted to the crisis intervention ward, 15.3% were admitted to the day clinic (where no coercive interventions can be performed and no violent acts happened, underlining the non-dangerous character of this population), and 28.9% were admitted to other wards of the hospital. Whilst the majority of patients were treated on the crisis intervention ward, only nine of the 24 coercive interventions and 15 of 29 aggressive acts occurred on this ward. Patients on the crisis intervention ward were significantly more frequently female, were younger and had a significantly shorter length of stay, no differences were found regarding clinical global impression at admission and discharge, number of previous admissions and marital status. Thus, even though the distribution of patients on the wards was certainly not random, the effects of the new structure are underestimated rather than overestimated by our evaluation of the entire hospital population.

## Discussion

The results provide a clear picture that the introduction of a crisis intervention ward had been successful on several levels. Firstly, according to the primary objective of the intervention, the number of coercive measures of any kind was significantly reduced. This demonstrates that a policy to reduce coercive interventions should aim at different targets – not only training, culture, and attitudes can be a successful strategy, but also focusing on the needs of different groups of patients. There is some evidence that systematic implementation of psychotherapeutic elements such as groups with cognitive-behavioural therapy or relationship management therapy can help to reduce coercive interventions [18,19]. In contrast to such interventions described before, we did not introduce single psychotherapeutic interventions but a different organisational structure as a whole.

Secondly, the decrease of coercive interventions was not paid for by an increase of violent acts. On the contrary, even violent behaviours decreased, first of all violence against persons. The decrease of violence and coercive interventions seems to be a robust result, because the evaluation was done conservatively. It took into account data from the whole hospital, including those patients who were still admitted to other acute admission wards, where coercive interventions happened more frequently than on the crisis intervention ward. Thirdly, the proportion of involuntarily treated patients significantly dropped from 3.8% to 0.6% among adjustment disorders and from 11.0% to 3.8% among personality disorders, which means a 70% reduction from 6.9% to 2.1% in the whole population. Data from another concomitant research project suggests that this is probably due to a ward atmosphere which is more appropriate for these patients than the atmosphere of an acute admission ward [20]. Thus patients can be motivated more easily to stay on a voluntary basis. In our opinion, this is the principal reason why the length of stay increased for people with adjustment disorders, admitted most frequently after a suicide attempt: Not being treated among psychotic and manic patients on a mixed admission ward, they feel better accepted with their specific problems in a therapeutic atmosphere and consent to stay there for a while on a voluntary basis.

Forthly, the advantages for both patients and staff were not at any cost to somebody else. The crisis intervention ward had not received additional staff for its new purpose. There was a mild increase of admissions, nearly exclusively women with borderline personality disorder. Whether this is a positive or a lamentable effect is an open question and needs further observation. The frequency of suicide attempts and self-damaging behaviour could not be reduced, which is a challenge for the future.

There are some limitations referring to the evaluation of the project. It is based on a pre-post-design, in absence of a control group. Thus it is only a descriptive study with limited conclusions. However, nearly all knowledge about the effects of organisational interventions stems from such observational studies, since genuine control groups are mostly impossible to achieve for such interventions. During the study period, the staff members were not aware that such a study would be conducted. Therefore it is not probable that they strongly tried to avoid coercive interventions in order to obtain good results. Further, the data used here is based on a routine documentation of therapists and staff and not on the records of independent researchers. This procedure has weaknesses with respect of the quality of data, but also has certain strength: The method can be performed legally in Germany as an aspect of quality assessment without informed consent of every individual patient. It is its advantage that it covers the full range of admitted patients without any selection. Staff are already being obliged to document each coercive action carefully in a standardized form. Thus the data has been completely available for evaluation and there is no evidence of any bias in this respect [12]. The data on aggressive and suicidal behaviour, however, stems from the basic documentation which has to be filled in by the responsible doctor and is possibly somewhat weaker in quality, hence its availability only in about 80% of the admissions (see table 1 and 2). No systematic bias for missing data was noticed.

To our knowledge, such a project which has been specifically designed to reduce coercive interventions for patients with personality and adjustment disorders in case of an acute crisis, has never been described before. Introducing a crisis intervention ward is not possible everywhere. The predisposition is a sufficient number of available hospital beds that allows dedication of one ward to the care of these patients.

## Conclusion

Two lessons can be learned and can stimulate future clinical ideas and research. Firstly, strategies to reduce coercive interventions should not always focus on all psychiatric patients but should take into account the different needs of different patient groups. Secondly, successful interventions can take place on the level of the individual patient as well as on the level of staff education, of hospital policy and of ward organisation and admission policy.

## Consent

This study was done as a project of quality management. Hospitals in Germany are legally obliged to do quality management and no informed consent is required as long as the data used is recorded in routine practice in the respective hospital and data is anonymously reported.

## Competing interests

The authors declare that they have no competing interests.

## Authors' contributions

TS developed the study design and the methodology and wrote most of the manuscript. ST and UG were responsible for the development of the specific elements of the intervention and the complete documentation of the data. CU was responsible for the data presentation (results section). FE was responsible for the calculation of data. PS was responsible for the selection and application of statistical methods. All authors made contributions to the phrasing of the manuscript.

## References

[B1] Fisher WA Restraint and seclusion: a review of the literature Am J Psychiatry 1994 151 11 1584 1591 7943445 10.1176/ajp.151.11.1584

[B2] Needham I Abderhalden C Coercive procedures and facilities in Swiss Psychiatry Swiss Medical Weekly 2002 132 253 258 12148079 10.4414/smw.2002.09926

[B3] Sailas E Fenton M Seclusion and restraint for people with serious mental illnesses Cochrane Database Syst Rev 2000 2 CD 001163 10.1002/14651858.CD001163 PMC6669266 10796606

[B4] Salib E Ahmed AG Cope M Practice of seclusion: a five-year retrospective review in North Cheshire Med Sci Law 1998 38 321 327 9808944 10.1177/002580249803800408

[B5] Martin V Bernhardsgrütter R Goebel R Steinert T The use of mechanical restraint and seclusion in patients with schizophrenia: A comparison of the practice in Germany and Switzerland Clin Pract Epidemol Ment Health 2007 3 1 17274830 10.1186/1745-0179-3-1 PMC1797172

[B6] Steinert T Gebhardt RP Erfolgen Zwangsmaßnahmen willkürlich? Psychiat Prax 2000 27 282 285 11050734

[B7] Le Bel J Stromberg N Duckworth K Kerzner J Goldstein R Weeks M Harper G La Flair L Sudders M Child and adolescent inpatient restraint reduction: a state initiative to promote strength-based care J Am Acad Child Adolesc Psychiatry 2004 43 37 45 14691359 10.1097/00004583-200401000-00013

[B8] Janssen WA Noorthoorn EO de Vries WJ Hutschemeakers GHM Lenemeijer HHGM Callahan P, Nijman H, Palmstierna T, Oud N The use of seclusion in the Netherlands compared to countries in and outside Europe Proceedings of the 5th European Congress on Violence in Clinical Psychiatry 2007 Amsterdam: Kavanah 164 166

[B9] Bowers L Nijman H Allan T Simpson A Warren J Turner L Prevention and management of aggression training and violent incidents on U.K. acute psychiatric wards Psychiatr Serv 2006 57 1022 1026 16816288 10.1176/ps.2006.57.7.1022

[B10] Richter D Whittington R and Richter D Non-physical conflict management and de-escalation Violence in Mental Health Settings: Causes, Consequences, Management 2006 New York: Springer 125 144

[B11] Gaskin CJ Elsom SJ Happell B Interventions for reducing the use of seclusion in psychiatric facilities. Review of the literature Br J Psychiatry 2007 191 298 303 17906239 10.1192/bjp.bp.106.034538

[B12] Steinert T Martin V Baur M Bohnet U Goebel R Hermelink G Kronstorfer R Kuster W Martinez-Funk B Roser M Schwink A Voigtländer W Diagnosis-related frequencies of compulsory measures in 10 German psychiatric hospitals and correlates with hospital characteristics Soc Psychiatry Psychiatr Epidemiol 2007 42 140 145 17180296 10.1007/s00127-006-0137-0

[B13] Steinert T Bohnet U Eisele F Martin V Martinez-Funk B Roser M Voigtländer W Freiheitseinschränkende Zwangsmaßnahmen bei Patienten in psychiatrischen Krankenhäusern. Epidemiologie und Qualitätsaspekte Der Nervenarzt 2006 12 1539 1544

[B14] Björkdahl A Heilig M Palmstierna T Hansebo G Changes in the occurrences of coercive interventions and staff injuries on a psychiatric intensive care unit Arch Psychiatr Nurs 2007 21 5 270 277 17904484 10.1016/j.apnu.2007.06.007

[B15] Abderhalden C Needham I Dassen T Halfens R Haug HJ Fischer J Predicting inpatient violence using an extended version of the Brøset-Violence-Checklist: instrument development and clinical application BMC Psychiatry 2006 6 17 16638122 10.1186/1471-244X-6-17 PMC1459151

[B16] Stone MH Management of borderline personality disorder: A review of psychotherapeutic approaches World Psychiatry 2006 5 15 20 16757985 PMC1472266

[B17] Cording C Gastpar M DGPPN-Empfehlung zur psychiatrischen Basisdokumentation [BADO] [DGPPN recommendation for basic psychiatric documentation] Der Nervenarzt 1997 68 930 931 9732744

[B18] Veltro F Falloon I Venditelli N Oricchio I Scinto A Gigantesco A Morosini P Effectiveness of cognitive-behavioural therapy for inpatients Clin Pract Epidemol Ment Health 2006 2 16 16859548 10.1186/1745-0179-2-16 PMC1552055

[B19] Hoch JS O'Reilly RL Carscadden J Relationship management therapy for patients with borderline personality disorder Psychiatr Serv 2006 57 179 181 16452694 10.1176/appi.ps.57.2.179

[B20] Uhlmann C Steinert T Stationäre Krisenintervention in der psychiatrischen Regelversorgung: Stationsatmosphäre und Behandlungserfolg nach Eröffnung einer Spezialstation für Persönlichkeitsstörungen [In-patient crisis intervention in standard psychiatric care: Ward atmosphere and outcomes after implementing a specialised ward for personality disorders] Verhaltenstherapie 2008 in press

